# Ratcave: A 3D graphics python package for cognitive psychology experiments

**DOI:** 10.3758/s13428-019-01245-x

**Published:** 2019-05-06

**Authors:** Nicholas A. Del Grosso, Anton Sirota

**Affiliations:** Bernstein Centre for Computational Neuroscience, Graduate School of Systemic Neurosciences, Faculty of Medicine, Ludwig-Maximillians-Üniversität München, Großhaderner Straße 2, 82152 Planegg, Germany

**Keywords:** 3D graphics, Python, Stimulus software, Vision, 3D

## Abstract

We present here a free, open source Python 3D graphics library called Ratcave that extends existing Python psychology stimulus software by allowing scientists to load, display, and transform 3D stimuli created in 3D modeling software. This library makes 3D programming intuitive to new users by providing 3D graphics engine concepts (Mesh, Scene, Light, and Camera classes) that can be manipulated using an interface similar to existing 2D stimulus libraries. In addition, the use of modern OpenGL constructs by Ratcave helps scientists create fast, hardware-accelerated dynamic stimuli using the same intuitive high-level, lightweight interface. Because Ratcave supplements, rather than replaces, existing Python stimulus libraries, scientists can continue to use their preferred libraries by simply adding Ratcave graphics to their existing experiments. We hope this tool will be useful both as a stimulus library and as an example of how tightly-focused libraries can add quality to the existing scientific open-source software ecosystem.

**Electronic supplementary material:**

The online version of this article (10.3758/s13428-019-01245-x) contains supplementary material, which is available to authorized users.

Cognitive psychology and neuroscience experiments use software that presents stimuli to a subject, detects subject responses, and logs events for future analysis, all with high temporal accuracy. An ever-expanding list of other features included in this software are compatibility with third-party hardware devices (e.g. button boxes, amplifiers, eye tracking systems), support for custom experimental designs, and online analysis for adaptive stimulus sequences; these tools are available both as self-enclosed software solutions (e.g. Neurobs Presentation, BCI2000, SuperLab, E-Prime) and open-source libraries (e.g. Psychtoolbox by Brainard, 1997; PsychoPy by Peirce, 2007; VisionEgg by Straw, 2008; Expyriment by Krause & Lindemann, 2013; for a review of psychophysics libraries, see Kötter, 2009). However, these popular libraries are missing 3D graphics support, needed for a wide range visual psychophysics experiments, such as 3D mental rotation or object recognition, virtual reality in spatial navigation research, to name a few. While 3D graphics libraries do exist in Python (e.g. Panda3D, PyOgre, Vizard) and other languages (e.g. Unity3D, Unreal Engine), the stimuli, logging, and hardware support of all of these libraries are designed to work with the windows and event loops they supply, making it difficult to integrate 3D graphics functionality into different psychophysics libraries without (sometimes-extensive) modification (e.g. to mix PsychoPy’s DotStim and Expyriment’s video support). In practice, this means that each software suite is relatively self-contained; researchers who require 3D stimuli, for example, have to, thereby, resort to use or develop different experiment control software when employing 3D visual stimuli (essentially, building interface to 3D game engines), losing out on the rich features that exist in the psychophysics software ecosystem developed for the 2D graphics. Extension libraries help reduce these feature-tradeoff decisions; for example, OpenSesame, a Python-powered GUI (Mathôt & Theeuwes, 2012), uses PsychoPy, Expyriment, and PyGame as “backends” to its experiment-building graphical interface, thereby supporting all researchers who rely on those libraries. A similar extension approach could be used for 3D stimuli--not to compete with the existing 3D frameworks on a feature-by-feature basis, but to simply add simple-to-use 3D stimulus presentation and manipulation support to the feature list of existing 2D stimulus libraries in Python.

In this paper, we present an open-source, cross-platform Python library called Ratcave that adds 3D stimulus support to all OpenGL-based 2D Python stimulus libraries, including VisionEgg, Psychopy, Pyglet, and PyGame. We review the core features of Ratcave (https://github.com/ratcave/ratcave) and highlight key connections of its interface to underlying graphics programming strategies (a thorough manual, complete with API guide and tutorials for first-time users can be found at https://ratcave.readthedocs.org). This library, which derives its name from our high-speed RatcaveVR experimental setup (Del Grosso, Graboski, Chen, Hernández, & Sirota, 2017), is designed to increase accessibility of 3D graphics programming to the existing ecosystem of psychology software for Python.

## Software description

### Built-in primitives and graphics resources

**Fig. 1:**
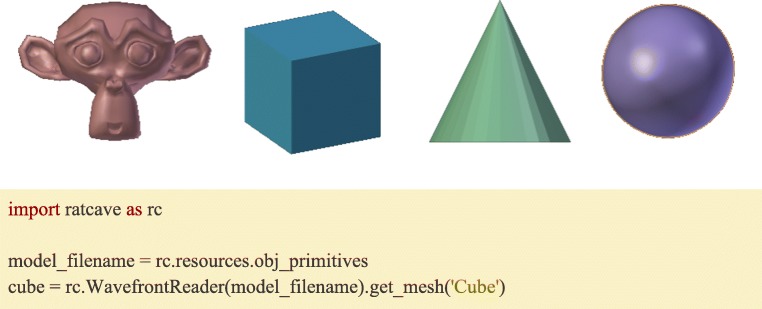
(Top): Some of the 3D Mesh primitives supplied with Ratcave. (Bottom): Importing Ratcave into the Python environment and creating a cube stimulus from the Ratcave’s supplied primitive meshes

**Fig. 2:**
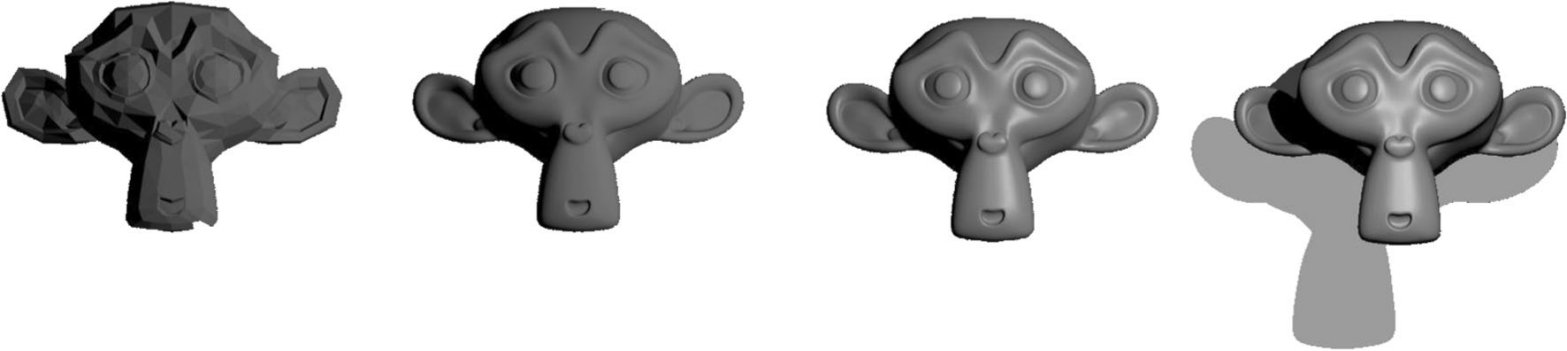
Examples of some simple 3D lighting effects available in Ratcave: diffuse and “glossy” specular reflections, ambient lighting, and shadows


In order to make 3D programming accessible, Ratcave comes with a collection of resources, including basic 3D object primitives (Fig. 1) and a wide range of 3D lighting effects (Fig. 2, Supplementary Video 1). This way, a user can get started quickly, writing customized code only when needed.

3D Lighting Cues. In this video, a 3D mesh has a series of lighting shaders applied to it to illustrate their importance in depth, scene, and motion perception: Diffuse shading, Specular highlights, shadow mapping, and image texture mapping. (AVI 26944 kb)


**Fig. 3:**
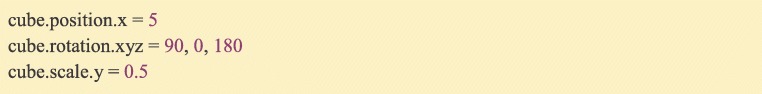
Code example: positioning, rotating, and scaling a Ratcave Mesh by assigning new values to their correspondingly-named attributes

Creating Meshes, a term used for any 3D object in Ratcave, is done either by supplying the vertex coordinates as an array or by importing from a 3d-formatted file (for example, the popular Wavefront file format, for which Ratcave provides a parser). All objects in a Ratcave Scene (Meshes, Lights, and Cameras) can be repositioned, rotated, and scaled using an intuitive object-oriented interface (Fig. 3).

### Rendering 3D Meshes in Ratcave

**Fig. 4:**
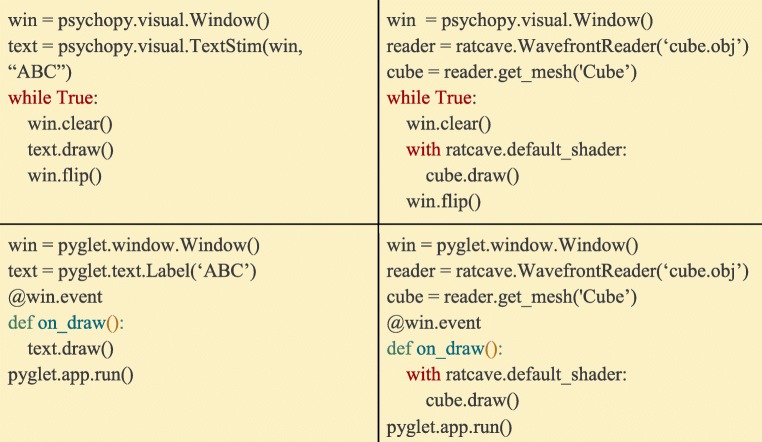
Demonstrating the flexibility and generality of Ratcave by drawing a cube in two different libraries: Psychopy (top) and Pyglet (bottom). On the left side is the code for drawing a 2D text stimulus for that library, with the right side showing the addition of a Ratcave cube stimulus. Although the syntax of each library differs from each other in how they create a window and begin a draw loop, the Ratcave drawing code is the same and inserts cleanly into the existing code structure for each experiment

Once a Mesh is loaded and positioned, it can be drawn in any active OpenGL window (e.g. a Psychopy window, Pyglet window, Vision Egg window, etc) by binding it to a Shader program using Python’s *with* keyword and calling its *draw()* method. Ratcave provides a default shader that performs many industry-standard 3D transformation and lighting steps (including diffuse and specular lighting, and shadow-mapping, Fig. 2), allowing users to create and use arbitrary 3D stimuli in syntactically the same way as they would use 2D stimuli (Fig. 4).

### Manipulating perspective in 3D scenes: Ratcave’s camera class

**Fig. 5:**
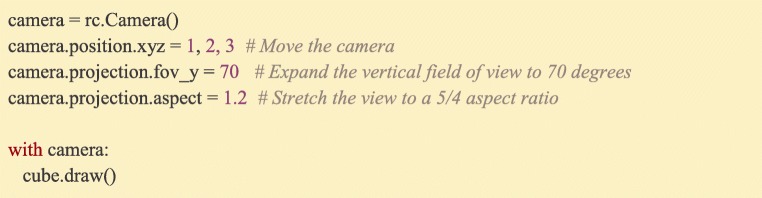
Positioning a Ratcave Camera is done using the same interface as with Mesh objects, and applying it to a draw operation which is done with the same interface as Shader objects. Changing the Camera’s intrinsic projection properties (e.g. field of view, aspect ratio, frustrum section, orthographic vs perspective projection) is done through its projection attributes

Unlike in 2D graphics, where the screen’s pixels provide a natural coordinate space for positioning objects, a 3D scene is composed of 3D objects (“Meshes”) viewed from a given perspective (the “Camera”) which is projected down onto the 2D surface of the display. Positioning objects on-screen is further made intuitive by Ratcave’s Camera class, which functions similarly to virtual cameras in 3D modeling software. Besides being positioned and rotated to face an object, properties of the Camera’s intrinsic projection model (e.g. field of view, aspect ratio, and frustrum cutoff thresholds, orthographic vs perspective projection) can be manipulated as well. To draw a Mesh from the perspective of the camera, it is bound by the user using a similar method as with the shader: using Python’s *with* keyword statement (Fig. 5).

### Working with groups of Meshes: scenes and scene graphs

**Fig. 6:**
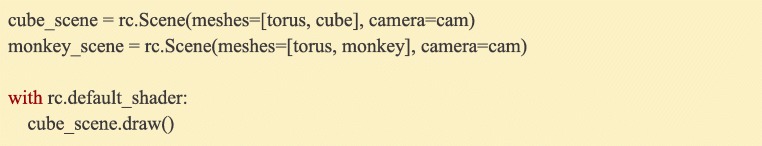
Collecting Meshes in a scene. Because scene objects hold Meshes, cameras, and lights, every Mesh they contain can be drawn in a single Scene.draw() call

Once all meshes are all loaded, they can be collected together in any Python iterator object and passed to a Scene, which is drawn using a draw() method. Scenes contain Mesh, Camera, and Light objects, which are applied automatically within the draw() call. Scenes can share objects between each other, making them useful, lightweight containers for different experimental conditions (Fig. 6).

**Fig. 7:**
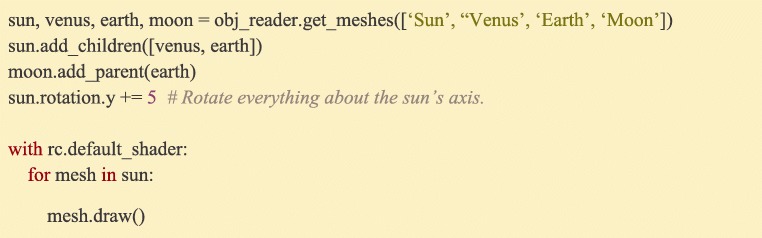
Building a scene graph. Meshes can be arranged in a tree-like parent/child collection using their add_children() and add_parent() methods, which sets their position and rotation to be relative to the parent. This tree can also be traversed by iterating over the top-most parent can then iterated over in a for loop

Complex relationships of object positions can be specified via Ratcave’s simplified scene graph functionality by parenting objects to each other, allowing the experimenter to move sets of objects in a single call to the top-most parent. For example, a much-simplified solar system model could be arranged as follows (Fig. 7).

### Integrating multiple rotation coordinate systems

Unlike 2D objects, there are multiple ways to format rotations in three-dimensional space, three of which are: euler rotations, rotation matrices, and quaternions. Euler rotations, sequential 2D rotations about three axes stored as XYZ coordinates, have the advantage of being intuitive to use and to set; a rotation about the X axis can be written as an angle in the X rotation coordinate. However, they also come with disadvantages; for example, they must be applied in the same order every time to achieve the same ending rotation (so mixing 3D modeling programs and 3D rendering programs in different order can result in unequivalent rotations), and they are vulnerable to a phenomenon known “gimbal lock”, a situation in which certain rotations can make a given axis useless. Rotation matrices, 3x3 square matrices that describe Euler rotations that apply rotation transformations with a single dot product, always apply the rotations in the same order as bound by the rules of linear algebra. However, building rotation matrices by hand is cumbersome at best. Quaternions, a single rotation about an arbitrary 3-element vector, stored as WXYZ or XYZW coordinates, have the advantage of being compact, non-sequential and invulnerable to gimbal lock; however, they can be unintuitive in practice. OpenGL also requires a fourth variant, a model matrix, that has a 4x4 matrix format, whose sequential application is order sensitive. Finally, different users may prefer setting their rotations as degrees or radians.

To allow full flexibility between different rotation coordinate systems, Ratcave allows all Physical object (Meshes, Cameras, and Lights) rotations to be set using any rotation coordinate system, as well as providing conversion methods between them: for example, to_quaternion(), to_euler(), and to_matrix(), with options in each for setting rotation sequence and radian or degree units. This feature is, naturally, optional; by default, all rotations are specified as Euler coordinates as degrees.

### Updating the data pipeline to the graphics card: uniforms, shaders, and vertex arrays

Ratcave uses modern OpenGL constructs from the ground up, rendering by passing data to graphics card-compiled “shader” programs, rather than sending individual commands to the OpenGL state machine from Python itself. While this creates a two-language situation (Python programs on the CPU and shader programs in the GLSL language on the graphics card) that may initially seem complex, it represents a scalable solution that allows scientists to take advantage of each language’s strengths. In addition, this approach helps with creating performant 3D graphics applications in slower dynamic languages like Python, where high numbers of C library calls (common in legacy OpenGL 3D applications) can create a significant performance bottleneck; in fact, this library is used by our lab’s virtual reality system to render full 3D scenes through a multi-pass rendering pipeline at 360 fps (data not shown). Three different types of data are passed to the graphics card, with each one wrapped by Ratcave with a Pythonic interface: Vertex Arrays, Uniforms, and Textures.

#### Vertex arrays

**Fig. 8:**
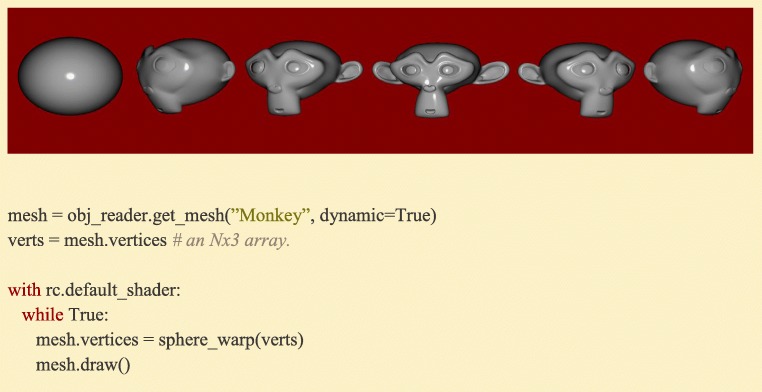
Warping a Mesh in real-time . If the Mesh is *dynamic* (meaning its data can be updated between frames), their Nx3 vertex array data can be accessed and manipulated like any NumPy array, and Ratcave will handle all graphics card buffering for the user. In this example sequence, a mesh is interpolated between its original coordinates (a Monkey primitive) and a sphere. For a video example, please see Supplementary Video 2


Meshes in 3D applications are composed of arrays of vertex coordinates, with each defining the endpoint of an edge or the boundary of a face on that Mesh. This data could be passed to the graphics card from Python point-by-point upon drawing (OpenGL’s ‘Immediate Mode’, used by many Python 2D graphics libraries), but this process can be made more efficient by sending the data as a single array using OpenGL’s VAO (Vertex Array Object) functionality and storing it on the graphics card itself. Sets of arrays (most commonly, a Mesh’s vertex, normal, and texture coordinate arrays) can be associated together via OpenGL’s VBO (Vertex Buffer Object), and then all that is needed is a single draw call when the actual rendering is performed. Since the data is already present on the graphics card, the operation is much more efficient. Ratcave pipes vertex array data using VAOs and VBOs on Meshes and uses pointers to associate NumPy arrays to the graphics card array data. The result is that users can pass NumPy arrays to Meshes and even edit them like normal NumPy arrays, while Ratcave updates the data on the graphics card as needed (Fig. 8). Using this approach, over 30,000 vertices can be streamed in real-time to the graphics card and rendered onscreen at 60 Hz, a performance level surpassing the needs of most behavioral research studies (Supplementary Video 2).

Real-Time Vertex Streaming. In this video, real-time dot stimuli generated in real-time are used to demonstrate the high computational performance level of Ratcave. Up to 30,000 dots are streamed and rendered at 60 frames per second. In addition, we demonstrate the simplicity of 3D transformations in 3D graphics engines like Ratcave lends itself to building and manipulating 3D structure-from-motion stimuli. (AVI 48194 kb)


#### Uniform data

**Fig. 9:**
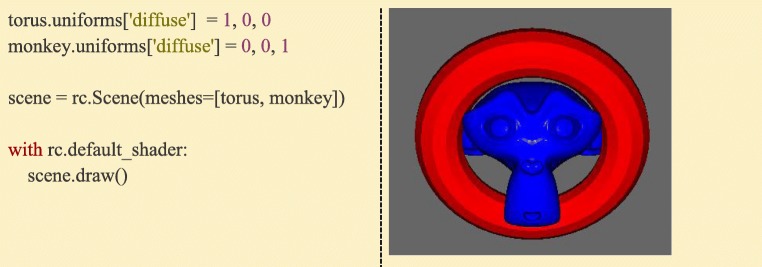
Creating and updating uniform values is done via the *uniforms* dictionary. In this example, updating the ‘diffuse’ property sends a 3-element vector to the shader upon drawing. If the shader program has a ‘diffuse’ variable declared, it will then use the supplied value. Ratcave’s default shader uses ‘diffuse’ to set the diffuse lighting color

Any data that can be associated with drawing a Mesh, whether its position, color, or even custom properties that are stimulus-specific can be received by the graphics shader as so-called “Uniform” data, meaning that it has the same value across all vertices of the Mesh. Uniform data can be single values, arrays, or even matrices. Most Ratcave objects have a dictionary-like *uniforms* attribute that automatically links, transforms, and sends its set of uniform data whenever the object is bound (whether by calling its *bind()* method or using Python’s *with* keyword) or drawn using a *draw()* method. Ratcave builds and maintains many uniforms automatically, including the matrices associated with positioning, viewing, and projecting objects on-screen (the Model matrix, which describes an object’s position; the View matrix, which describes the camera’s position; and the Projection matrices, which describes the camera’s lens characteristics) and adds some extra uniforms for coloring and lighting a mesh (Fig. 9).

**Fig. 10:**
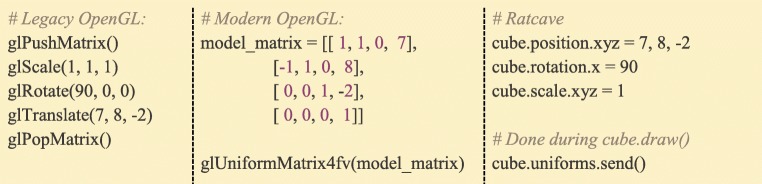
Comparison of Model Matrix computation and sending to OpenGL between legacy OpenGL, modern shader-based OpenGL, and Ratcave’s interface to modern OpenGL

Besides enabling full customizability of all stimuli, using uniforms helps increase performance of OpenGL rendering pipelines in Python. Legacy OpenGL typically requires five library calls to position an object on-screen, even if the mesh’s position is unchanged from the last rendered frame. As the number of objects to be rendered increases, the computational cost scales linearly. Utilizing shaders, on the other hand, requires only a single library call, which sends a single matrix (a “Model” matrix) to the shader. Ratcave makes calculating these matrices straightforward by automatically updating the model matrix whenever a stimulus’ position, rotation, or scale attributes are modified. It also saves these transformations intelligently, “lazily” updating the matrix (via an Observer software design pattern) only when needed, using the optimized Numpy array library. Sending the matrix to the shader is done when the “draw()” method is called. Similar steps are done for the Camera’s view matrix and projection matrix (Fig. 10).

#### Texture data

Ratcave also supports mapping image data to 3D meshes using a technique called “UV mapping”, named after the coordinate system used for specifying the rows and columns of an image (“u” and “v”). With this method, each Mesh’s vertex is associated with an image and its 2D coordinate (called a “texture” and a “texture coordinate”, respectively), and the image’s pixels are interpolated between the vertices, effectively stretching the 2D image across the Mesh’s surface.

**Fig. 11:**
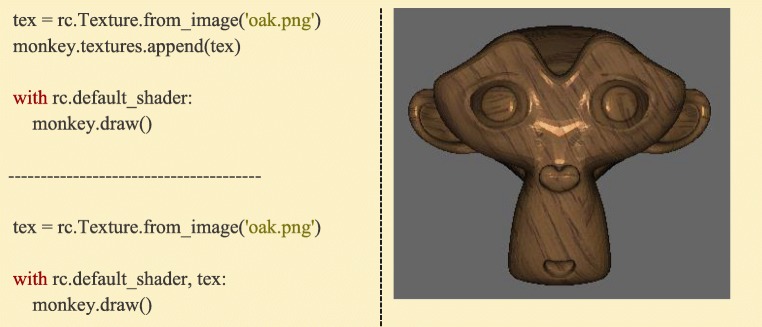
Images can be read in as OpenGL textures. To bind them, they can be either appended to Meshes in order to automatically activate them when the mesh is drawn (top-left), or activated explicitly with the *with* statement before drawing (bottom-left). Each approach has advantages for different experimental paradigms


The following three steps are needed to display this data using OpenGL: the texture data must be formatted and passed to the graphics card as an OpenGL Texture, it must be bound, and it must have an associated uniform name in order to link a given texture with a given rendering step in the shader. All of these steps are performed by Ratcave’s Texture objects, taking the OpenGL ID from an image loaded using another image processing library (making it compatible with a wide variety of image processing software), or loading it from an image file using Pyglet’s image module. If it is appended to a Mesh’s textures list attribute, it is automatically bound and its uniforms sent upon the Mesh’s *draw()* method call. Any number of textures of any OpenGL type (e.g. color vs depth textures, 2D vs 3D Textures, 2D vs Spherical vs 3D texture coordinates) can be appended to a Mesh, allowing any image algorithm to be implemented on the graphics card online, during stimulus rendering (Fig. 11, Supplementary Video 3).

Deferred Rendering. In this video, we demonstrate both 2D stimuli (e.g. moving Gabor patches) and 3D stimuli (e.g. Shadow projections from an arbitrary light source position) created using OpenGL shader programs, highlighting the utility of shaders in stimulus libraries. (AVI 10841 kb)


### Writeable textures: building deferred rendering pipelines

**Fig. 12:**
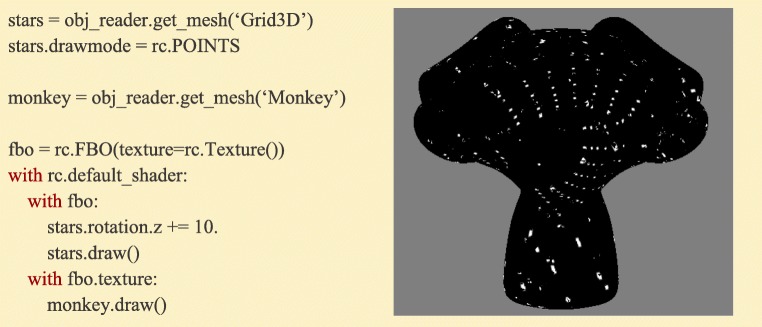
An example of two-pass rendering using Framebuffer objects. First, the stars Mesh is drawn and the resulting image saved to a Framebuffer-attached texture. This texture then becomes the texture bound to the monkey Mesh, producing an unusual effect: a rotating star field mapped on a monkey head

OpenGL’s Framebuffer objects allow users to create virtual windows that redirect a rendered image to a texture saved in memory on the graphics card instead of the display. This creates opportunities to build “deferred” rendering pipelines, in which several different image processing algorithms are run and saved for a final step that combines the previous images into more complex and dynamic images (Fig. 12).

Deferred rendering is an important technique for CAVE-style virtual reality systems, which project a 360-degree rendering of the virtual environment onto projection screens surrounding the subject (Del Grosso et al., 2017). This is done in two steps: first, the full virtual environment is saved to six textures, each representing a different viewpoint of the scene from the perspective of the subject. Then, these textures are drawn onto meshes representing the screen from the perspective of the video projector and rendered onscreen. When all of these renders are counted up, a total of seven rendering passes (six renders-to-texture and one render-to-screen) are performed. Deferred rendering is also useful for rendering shadows, where the scene is first rendered to texture from the perspective of a light source in order to calculate where the shadow should appear from the camera’s perspective for the final render-to-screen pass. Any multi-pass rendering algorithm can be done in Ratcave by simply binding an FBO object and then drawing a scene (Fig. 2).

### OpenGL shader programs

**Fig. 13:**
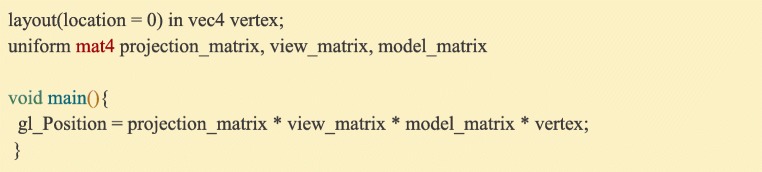
A GLSL Vertex Shader. This program takes the vertex data (the first array in a Ratcave mesh, specified as location 0) and the various matrices (received as uniforms), and calculates the onscreen position by calculating their dot product, outputting the onscreen position

**Fig. 14:**
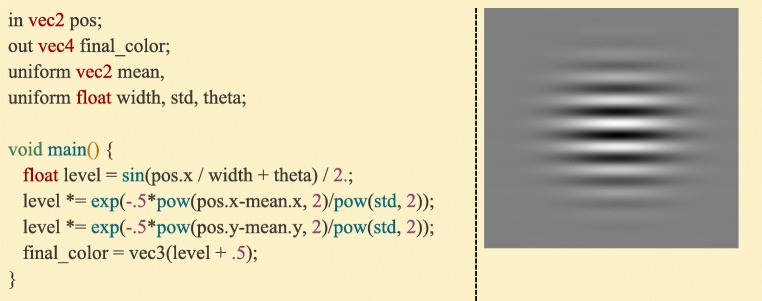
A GLSL Fragment Shader. This program is run for every pixel where a mesh is present. It takes uniform data (“width”, “mean”, etc) and outputs the RGB (“final_color”). This program calculates a gabor patch based on the screen position of a pixel (‘pos’) and the uniform parameters given by the Ratcave program

Besides enabling full customization of graphics rendering, OpenGL’s programmable pipeline speeds up graphics applications by allowing users to off-load calculations to the graphics card through “shader” programs written in a C-like language called GLSL. Each program is made up of two smaller programs: one that is run for each mesh vertex (the “Vertex Shader”, Fig. 13), which is most commonly used for positioning something onscreen, and one that is run for each pixel of the display (the “Fragment Shader”, Fig. 14, Supplementary Video 3), which is most commonly used for setting the color and lighting properties of the image.

**Fig. 15:**
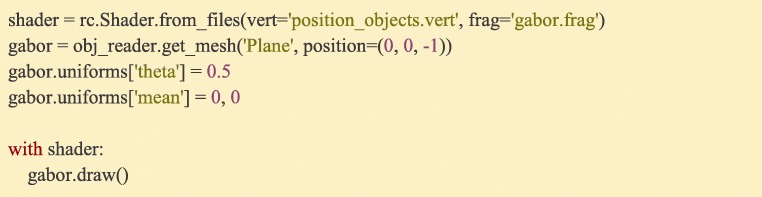
Loading custom shader files into a Ratcave Shader object and using it to draw a stimulus. Uniform values get sent to the shader when the draw() method is called, thereby connecting shader program variables to Python variables

Ratcave Shader objects compile these programs when needed and run them when bound, as seen in previous examples (Fig. 15). Because shader programs can be mixed and matched, and because they run on all hardware, platforms, and graphics engines, these short programs are useful formats for a wide variety of visual stimuli.

## Discussion

Ratcave is useful for Python-using scientists who want both 3D graphics support in their existing experiment scripts and a quick introduction to computer graphics in OpenGL, smoothing the learning curve toward using more advanced, feature-complete 3D graphics software. Because Ratcave provides access to the more-advanced OpenGL rendering methods (e.g. framebuffers and custom shaders), researchers can also use Ratcave to rapidly prototype complex visual stimuli. These GLSL shader programs run directly on the graphics card (e.g. the Gabor patch stimulus in Fig. 14, often used for vision studies), making them cross-compatible between Python programmers using Ratcave, Matlab programmers using Psychtoolbox, and 3D graphics engine users (e.g. Unity3D, Unreal Engine 4, and Panda3D), a property that has interesting potential for future stimulus libraries.

Future development of Ratcave will include support for more 3D-object file formats, stereoscopic stimuli, animations, logging-event hooks, and more powerful scene graph functionality. Ratcave’s design also cleanly separates 3D object manipulation and GPU interaction, making it possible to extend support to other low-level graphics libraries (e.g. WebGL or Vulkan) through an adapter programming design pattern, should future psychology stimulus software in Python use these graphics libraries themselves.

With the extension package described in this paper, psychology researchers can add and manipulate 3D stimuli with minimal code in a familiar programming environment. Researchers can simply drop-in their 3D stimuli into experiment scripts that support their input hardware and experimental design managers. Ratcave is easy to use, and the most-used operations on 3D models (importing data from file, building a mesh, manipulating its position, rotation, and scale, change its lighting, and drawing to the screen) can be done with single lines of code. As such, it makes for a good addition to the existing Python psychology software ecosystem.

## Electronic supplementary material

ESM 1 (GZ 5053 kb)
