# Supplementary figures and images for: Ratcave: A 3D graphics python package for cognitive psychology experiments
Source: Behav Res Methods. 2019 May 6;51(5):2085–93. doi: 10.3758/s13428-019-01245-x (PMC6797704; doi:10.3758/s13428-019-01245-x)

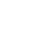

Supplement: Supplementary file 4 — (GZ 5053 kb) [file 13428_2019_1245_MOESM4_ESM.gz › ratcave-0.7.1rc2/assets/white.png]

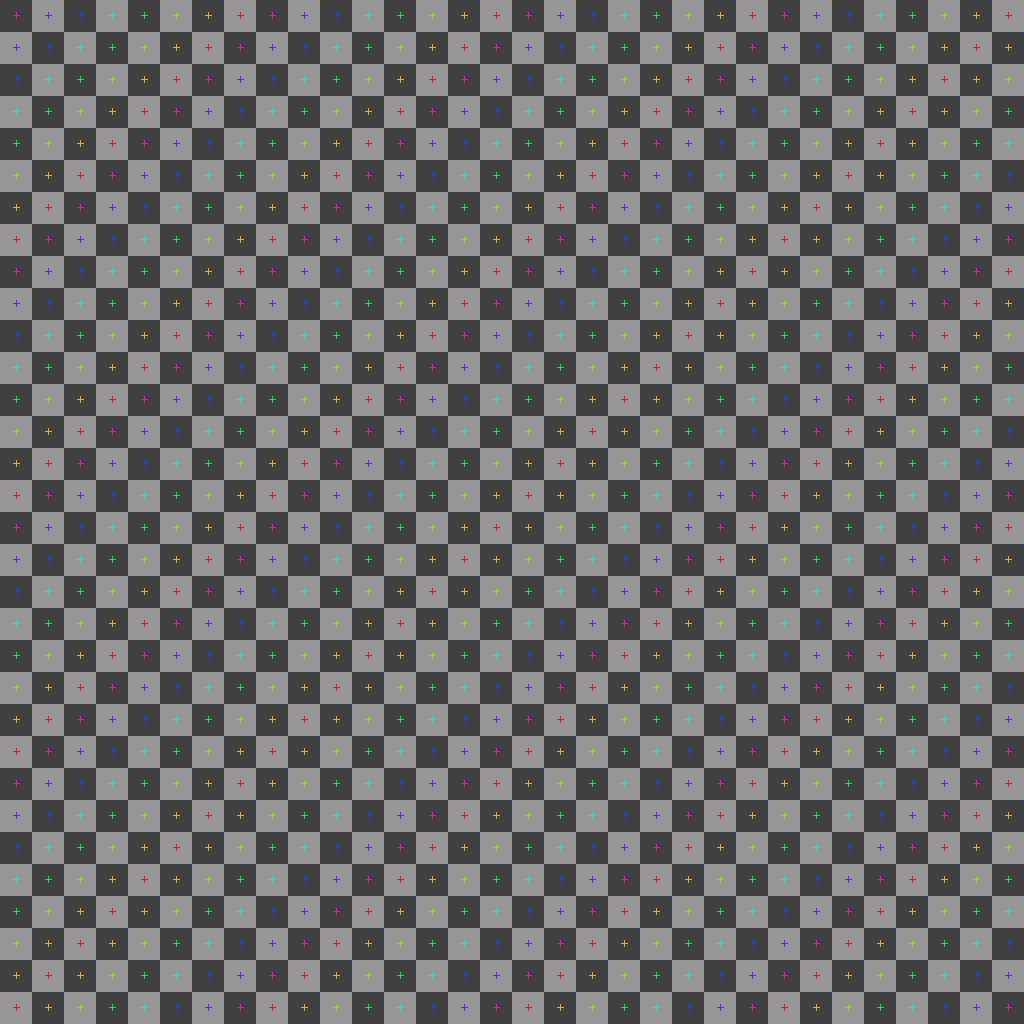

Supplement: Supplementary file 4 — (GZ 5053 kb) [file 13428_2019_1245_MOESM4_ESM.gz › ratcave-0.7.1rc2/assets/uvgrid.png]

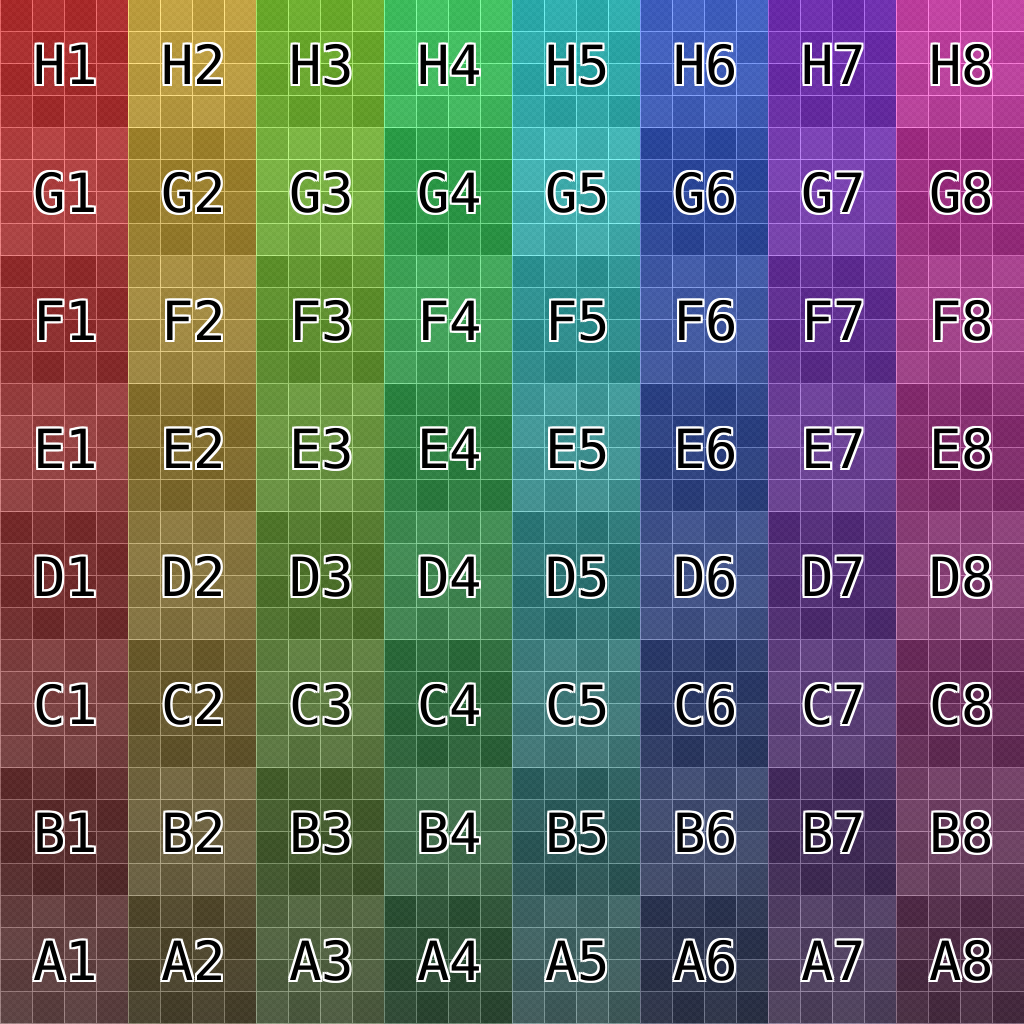

Supplement: Supplementary file 4 — (GZ 5053 kb) [file 13428_2019_1245_MOESM4_ESM.gz › ratcave-0.7.1rc2/assets/colorgrid.png]

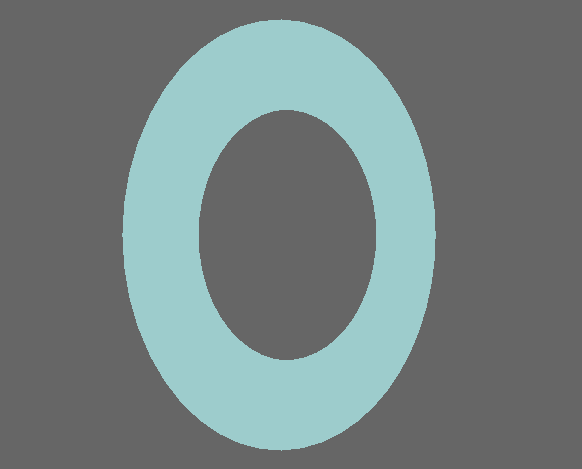

Supplement: Supplementary file 4 — (GZ 5053 kb) [file 13428_2019_1245_MOESM4_ESM.gz › ratcave-0.7.1rc2/docs/_static/tut3_blue_torus.png]

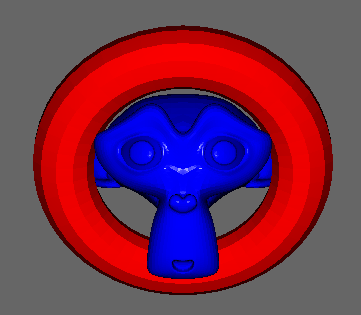

Supplement: Supplementary file 4 — (GZ 5053 kb) [file 13428_2019_1245_MOESM4_ESM.gz › ratcave-0.7.1rc2/docs/_static/redblue.png]

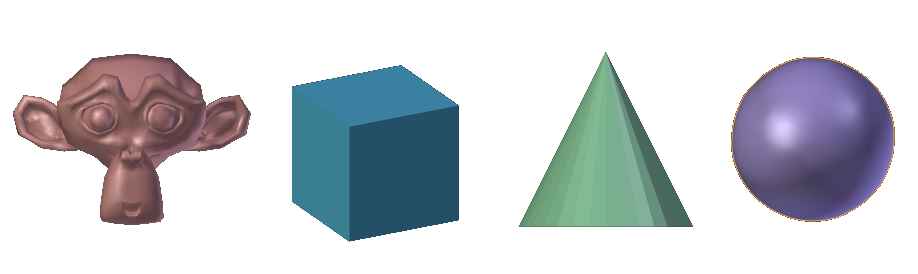

Supplement: Supplementary file 4 — (GZ 5053 kb) [file 13428_2019_1245_MOESM4_ESM.gz › ratcave-0.7.1rc2/docs/_static/primitives.png]

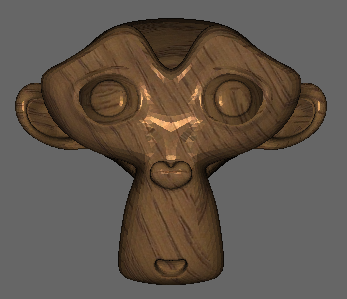

Supplement: Supplementary file 4 — (GZ 5053 kb) [file 13428_2019_1245_MOESM4_ESM.gz › ratcave-0.7.1rc2/docs/_static/wood_monkey.png]

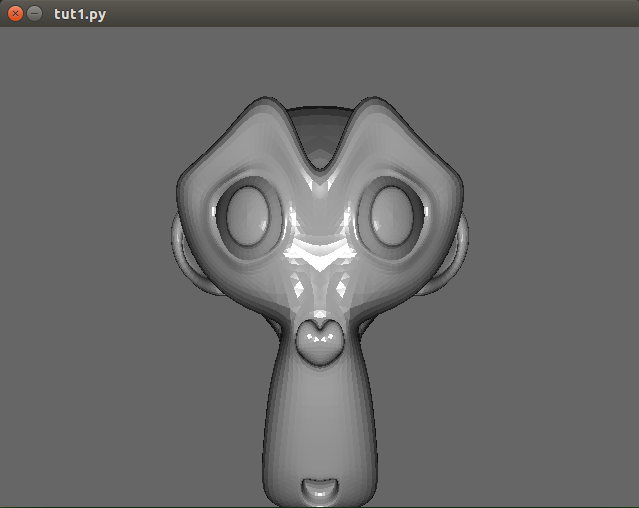

Supplement: Supplementary file 4 — (GZ 5053 kb) [file 13428_2019_1245_MOESM4_ESM.gz › ratcave-0.7.1rc2/docs/_static/tut1_gray_monkey.png]

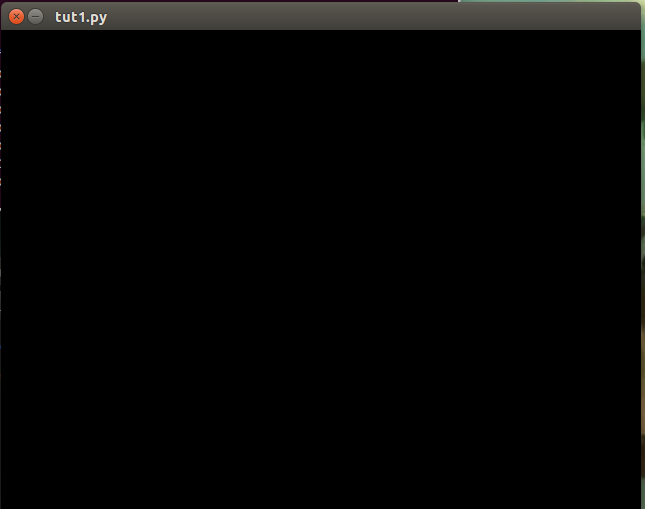

Supplement: Supplementary file 4 — (GZ 5053 kb) [file 13428_2019_1245_MOESM4_ESM.gz › ratcave-0.7.1rc2/docs/_static/tut1_blank_window.png]

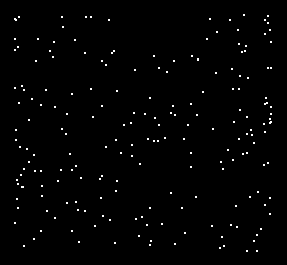

Supplement: Supplementary file 4 — (GZ 5053 kb) [file 13428_2019_1245_MOESM4_ESM.gz › ratcave-0.7.1rc2/docs/_static/dots.png]

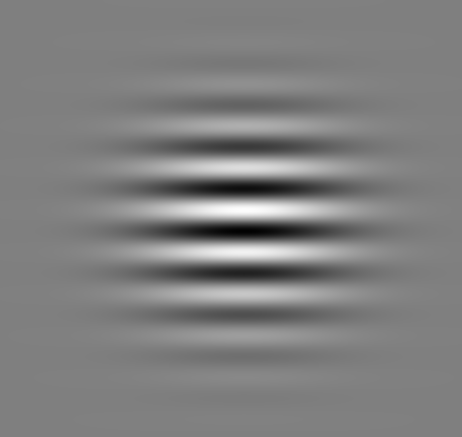

Supplement: Supplementary file 4 — (GZ 5053 kb) [file 13428_2019_1245_MOESM4_ESM.gz › ratcave-0.7.1rc2/docs/_static/gabor_patch.png]

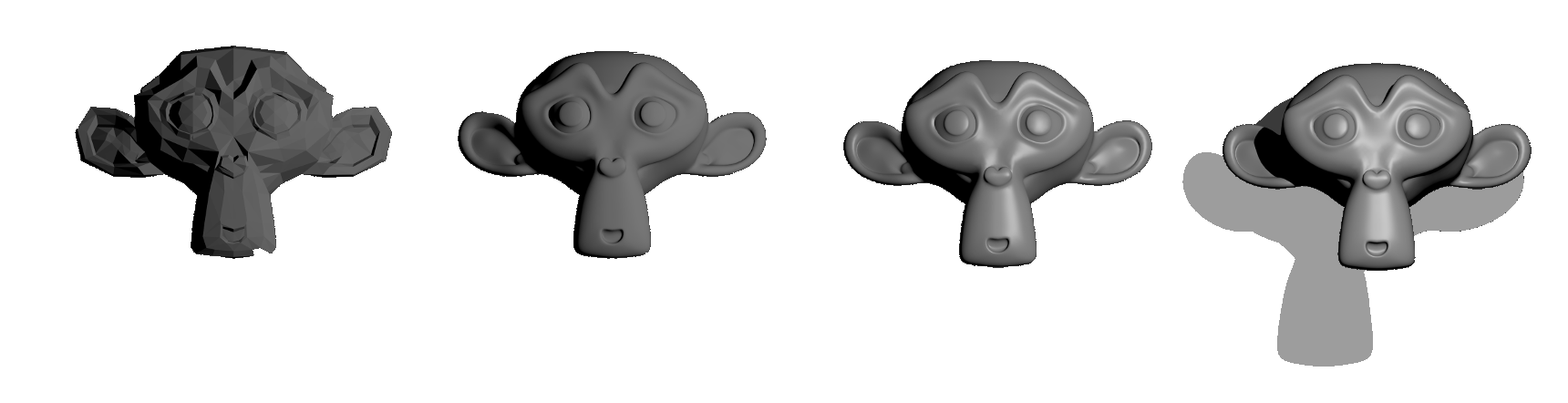

Supplement: Supplementary file 4 — (GZ 5053 kb) [file 13428_2019_1245_MOESM4_ESM.gz › ratcave-0.7.1rc2/docs/_static/shading_example.png]

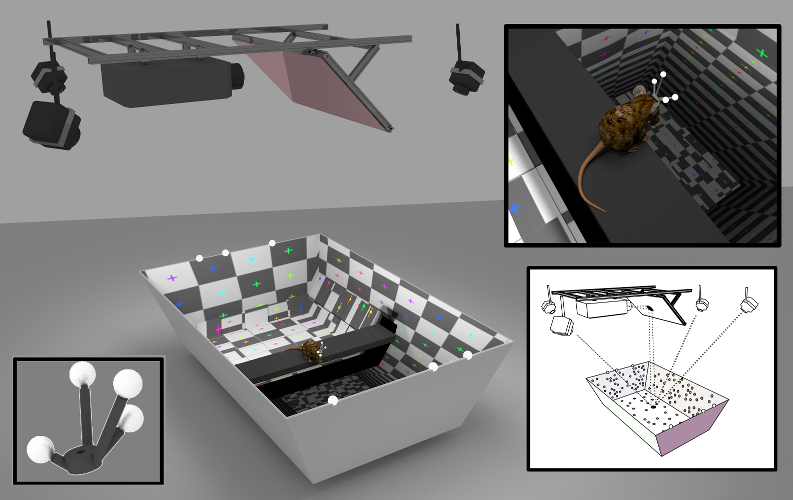

Supplement: Supplementary file 4 — (GZ 5053 kb) [file 13428_2019_1245_MOESM4_ESM.gz › ratcave-0.7.1rc2/docs/_static/finalvr_composite3_small.png]

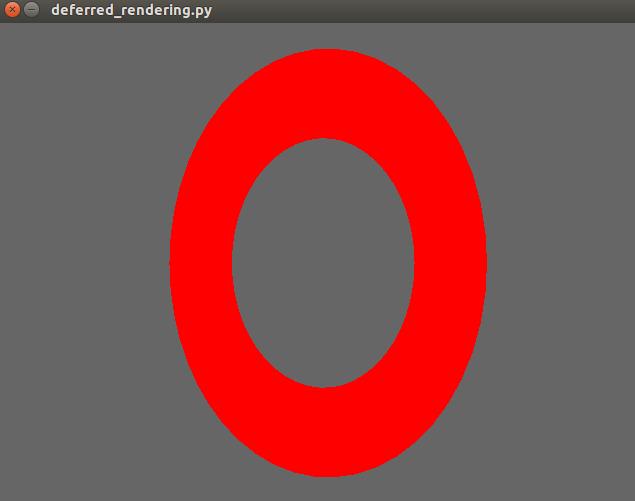

Supplement: Supplementary file 4 — (GZ 5053 kb) [file 13428_2019_1245_MOESM4_ESM.gz › ratcave-0.7.1rc2/docs/_static/tut3_red_torus.png]

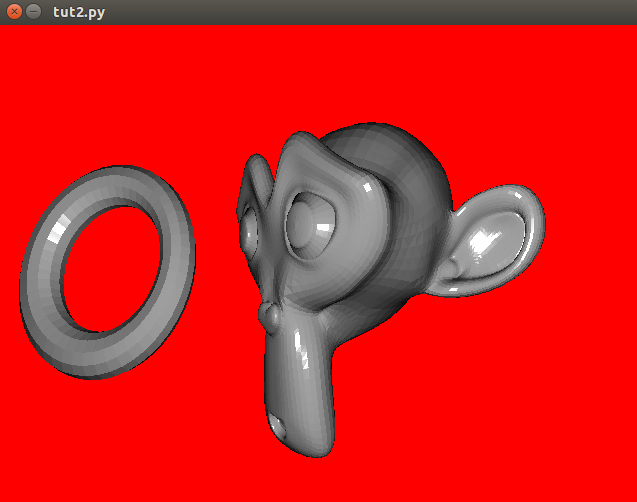

Supplement: Supplementary file 4 — (GZ 5053 kb) [file 13428_2019_1245_MOESM4_ESM.gz › ratcave-0.7.1rc2/docs/_static/tut2_final.png]

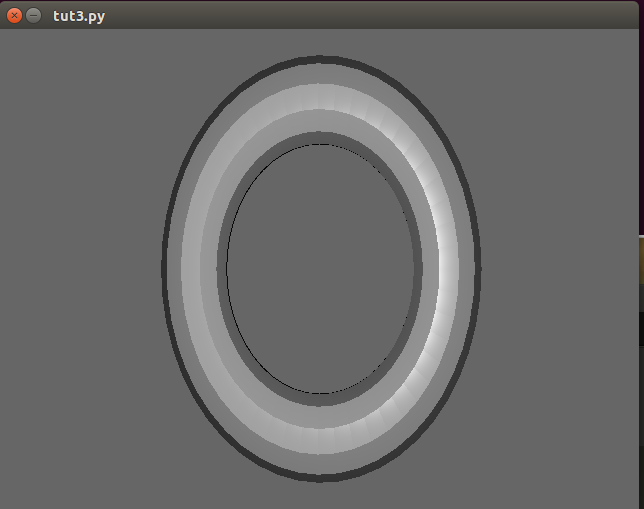

Supplement: Supplementary file 4 — (GZ 5053 kb) [file 13428_2019_1245_MOESM4_ESM.gz › ratcave-0.7.1rc2/docs/_static/tut3_gray_torus.png]

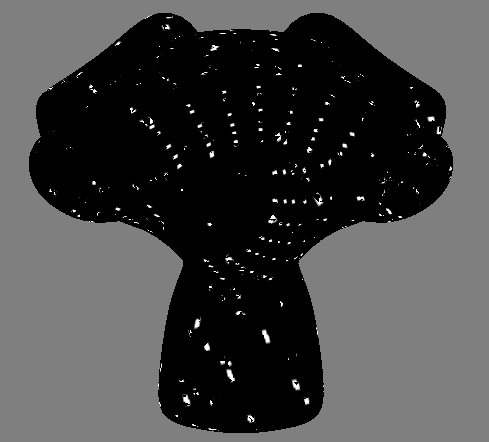

Supplement: Supplementary file 4 — (GZ 5053 kb) [file 13428_2019_1245_MOESM4_ESM.gz › ratcave-0.7.1rc2/docs/_static/monkey_starfield.png]

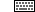

Supplement: Supplementary file 4 — (GZ 5053 kb) [file 13428_2019_1245_MOESM4_ESM.gz › ratcave-0.7.1rc2/htmlcov/keybd_closed.png]

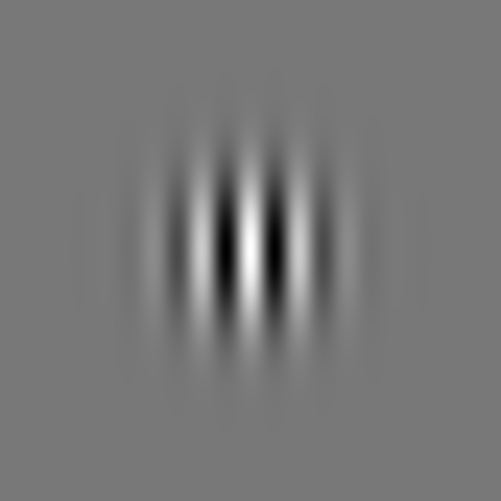

Supplement: Supplementary file 4 — (GZ 5053 kb) [file 13428_2019_1245_MOESM4_ESM.gz › ratcave-0.7.1rc2/examples/gabor-50-50.png]
